# Casein-assisted biomineralization of calcium carbonate microspheres for enhanced surface and adsorption properties

**DOI:** 10.3389/fbioe.2025.1654712

**Published:** 2025-10-07

**Authors:** Aniket Gade, Julia Nadrowska, Joanna Trzcińska-Wencel, Marek Wiśniewski, Rajesh Raut, Mahendra Rai, Patrycja Golińska

**Affiliations:** ^1^ Department of Microbiology, Faculty of Biological and Veterinary Sciences, Nicolaus Copernicus University in Toruń, Toruń, Poland; ^2^ Department of Biological Sciences and Biotechnology, Institute of Chemical Technology, Mumbai, India; ^3^ Department of Materials Chemistry, Adsorption and Catalysis, Faculty of Chemistry, Nicolaus Copernicus University in Toruń, Toruń, Poland; ^4^ Department of Botany, The Institute of Science, Mumbai, Dr. Homi Bhabha State University, Mumbai, India; ^5^ Department of Chemistry, Federal University of Piaui (UFPI), Teresina, Brazil

**Keywords:** biomineralization, calcium carbonate microsphere, casein, scanning electron microscopy, carriers

## Abstract

**Introduction:**

Biomineralization is a key biological process by which organisms form mineralized structures, with calcium carbonate being one of the most abundant naturally occurring biominerals. The development of synthetic analogs, particularly calcium carbonate microspheres (CaCO_3_-MS), holds potential for various applications, including as carrier materials.

**Methods:**

In this study, CaCO_3_-MS were synthesized using a precipitation method, both with and without casein. Ammonium, sodium, and potassium carbonate were evaluated as precipitating agents to optimize microsphere formation. The physical properties of the resulting microspheres were characterized using nitrogen adsorption analysis, Brunauer-Emmett-Teller (BET) analysis, diffuse reflectance infrared Fourier transform spectroscopy (DRIFT), scanning electron microscopy (SEM), transmission electron microscopy (TEM), and X-ray diffraction (XRD) analysis.

**Results:**

Ammonium carbonate was the most effective precipitating agent, yielding well-formed microspheres. Casein-assisted CaCO_3_-MS exhibited a higher specific surface area (65 m^2^/g) than CaCO_3_-MS synthesized without casein (47 m^2^/g). The casein-containing microspheres also demonstrated a more uniform spherical morphology, increased pore volume, higher surface energy, enhanced hydrophilicity, and approximately double the water adsorption capacity. However, both variants showed similar adsorption-desorption kinetics.

**Discussion:**

The presence of casein significantly improved the structural and functional properties of CaCO_3_-MS, making them more suitable for use as carrier materials. Furthermore, the described method enables the large-scale, surfactant-free synthesis of uniformly sized microspheres, enhancing its practical applicability.

## 1 Introduction

Biomineralization is a highly regulated and fundamental process where organisms produce minerals to harden or stiffen tissues, such as bones, shells, and teeth (Liu et al., 2016; Holzmeister et al., 2018) and provide support for soft tissue to make it rigid, provide sheltering and protection from prey, and breaking down food (Di Costanzo, 2022). These biominerals form under mild physiological pH, ambient pressure, and temperature conditions. Moreover, these biominerals exhibit higher mechanical strength than nonbiogenic minerals (Arakaki et al., 2015). Among the various biominerals, researchers most extensively study calcium carbonate (CaCO_3_) because it is the most abundant in nature and holds significant biological and environmental importance. The role of proteins in biomineralization has attracted significant interest, particularly in the formation of CaCO_3_ microspheres (CaCO_3_-MS), which have applications in materials science, biomedicine, *in vivo* imaging, and environmental science (Hu et al., 2024a). Organisms generally possess biominerals, which are often combined with proteins that act as binding agents, helping to hold together tiny inorganic crystals into larger biomineral aggregates. Apart from performing a significant role in crystal nucleation and mineral size regulation, proteins also provide properties that make them more elastic and resilient to pressure (Di Costanzo, 2022).

The preparation of CaCO_3_ of specific size and shape remains a challenge. The wide range of origins, compositions, morphologies, and polymorphic forms of CaCO_3_ makes it an essential material for both scientific study and technological use. Its importance has generated greater interest in researchers to fuel intensive research efforts aimed at synthesizing CaCO_3_ with precise control over its size, shape, crystal form, and surface characteristics. Formation of the CaCO_3_-MS by the precipitation method is a standard chemical process controlled mainly by factors like the concentration of calcium ions, the concentration of carbonate ions, pH, temperature, stirring time, stirring speed, and the availability of nucleation sites (Hammes and Verstraete, 2002). Although several established strategies have been reported for the synthesis of CaCO_3_-MS, such as templating, surfactant-assisted, and polymer-stabilized methods, these approaches typically suffer from limitations, including complex multistep procedures, high surfactant consumption, or difficulties in removing stabilizers. At the same time, our strategy in the present study provides a surfactant-free and environmentally benign route to achieve uniform CaCO_3_-MS with controlled morphology. It simplifies the synthesis and enhances reproducibility and scalability, highlighting our design’s novelty and practical significance. It is important to choose salt concentration, stirring time, and stirring speed properly because all these parameters influence the size of the CaCO_3_ microparticles. In addition to the control on the synthesis of particular shapes, sizes, and polymorphs of the synthetic CaCO_3_ microparticles, their surface functionalities are crucial for their applications as carrier molecules. Sodium caseinate, the sodium salt of the milk protein casein, is a naturally derived food additive known for its excellent emulsifying, foaming, and water retention properties, along with significant nutritional benefits (Hu et al., 2024b). The use of casein to form the MS has been reported by Voinescu et al. (2008), who described formation of novel hemispherical three component vaterite MS using alkaline silica, casein and diffusion of atmospheric carbon dioxide into the solution. Different modifications influenced the crystallization processes in casein structures. There is also a report of involvement of casein and magnesium ions in CaCO_3_ mineralization (Zhang et al., 2016). The concentration of casein significantly affects the morphology of CaCO_3_ crystals, and the secondary structure of casein proteins and the size of casein micelles play an important role in the formation of CaCO_3_-MS and its morphology. Li et al. (2017) reported the formation of stable vaterite CaCO_3_-MS by the fast precipitation method in the presence of only casein. They recommended that these MS could also be used as a drug carrier.

CaCO_3_-MS fabricated using casein shows attractive physical and chemical functional properties and great potential for encapsulating bioactive compounds such as drugs, nanoparticles, and dietary supplements. Moreover, casein has already demonstrated its potential as a carrier of biologically active agents (Głąb and Boratyński, 2017). We are also working on encapsulation of nanoparticles in CaCO_3_-MS, such that encapsulated nanoparticles will be released based on the stimulus, so that the controlled release of nanoparticles can be achieved. Therefore, this study aimed to evaluate the suitability of a carrier composed of casein and CaCO_3_-MS by analyzing its specific surface area, pore volume, porosity, and hydrophilicity, with potential applications in the delivery of drugs, nutrients, nanoparticles, and bioactive agents. This study systematically analyzed the preparation of CaCO_3_-MS via the precipitation method, focusing on carrier formation with and without casein.

## 2 Materials and methods

### 2.1 Materials

Procured the materials from the following sources: sodium caseinate salt (Glentham Life Sciences, Germany), sodium carbonate anhydrous (Na_2_CO_3_, Chempur, Poland), potassium carbonate (K_2_CO_3_, Chempur, Poland), ammonium carbonate ((NH_4_)_2_CO_3_, Chempur, Poland), citric acid monohydrate (Chempur, Poland), and calcium chloride (CaCl_2_·2H_2_O, Merck, Germany).

### 2.2 Preparation of CaCO_3_ microspheres

The preparation of CaCO_3_-MS with and without sodium caseinate was initiated by mixing calcium chloride and carbonate salts (Na_2_CO_3_, K_2_CO_3_, and (NH_4_)_2_CO_3_) solutions by precipitation method. Briefly, 20 mL of 10% carbonate salt solution was mixed with sodium caseinate and rapidly added to 10 mL of CaCl_2_ and 3 mL of 10% citric acid and thoroughly agitated on a magnetic stirrer (500 rpm) at room temperature.

After agitation, the reaction mixture was left undisturbed for 15 min to allow the formation of an amorphous primary CaCO_3_ precipitate, which gradually transformed into spherical MS. Finally, the precipitate was separated by centrifugation at 10,000 rpm for 10 min, washed with sterile water twice, and with absolute ethanol to remove any debris, and dried overnight by freeze drying and stored at room temperature.

The concentration of sodium caseinate and stirring time affected the size of the CaCO_3_-MS formed. To optimize the formation of smaller CaCO_3_-MS particles, various concentrations of sodium caseinate, i.e., 100, 200, 300, 400, 500, and 1,000 mg, were added to the reaction mixture, and stirring times of 2, 5, 15, 30, and 60 min at 500 rpm and room temperature were evaluated.

### 2.3 Characterization of microspheres

Electron microscopy, X-ray diffraction (XRD) and Fourier transform infrared (FTIR) spectroscopy. The freeze-dried casein-CaCO_3_-MS and CaCO_3_-MS powder was placed on a sample holder, followed by coating with nanogold with palladium using a mini sputter coater (SC7620, Quorum Technologies, United Kingdom), to generate the contrast, and analyzed using the high-resolution scanning electron microscope/focused ion beam hybrid instrument (Quanta 3D FEG, Fei, Hillsboro, OR, United States) for scanning electron microscopy (SEM). transmission electron microscopy (TEM), XRD, and FTIR were performed as described previously by Trzcińska-Wencel et al. (2023).

#### 2.3.1 Low-temperature N_2_ adsorption

The nitrogen adsorption isotherms were measured at 77.5 K using the Autosorp iQ gas adsorption apparatus (Quantachrome, United States) as previously reported by Staroń et al. (2024). Before measurement, the carbon samples were desorbed in a vacuum (below 10^−3^ Pa) at 323 K for 12 h.

#### 2.3.2 Hydrophilicity–H_2_O adsorption and kinetics of H_2_O desorption

A Nicolet iS50 FTIR spectrometer (Thermo Scientific, United States) with a Praying Mantis diffuse reflectance infrared Fourier transform spectroscopy (DRIFT) environmental chamber was used for analysis of hydrophilicity. We typically collected 32 scans at a resolution of 4 cm^-1^ in the 600 to 8,000 cm^-1^ range. Water adsorption was performed under isobaric conditions (p = 4 kPa by flowing Ar gas through an H_2_O scrubber at 25 °C). A Praying Mantis *in situ* cell from Harrick Scientific Corporation was used as a reactor for the DRIFT studies. The construction of this cell enables the thermal treatment of the powdered sample up to 600 °C in any controlled atmosphere or a vacuum (Guo et al., 2020).

## 3 Results and discussion

**FIGURE 1:**
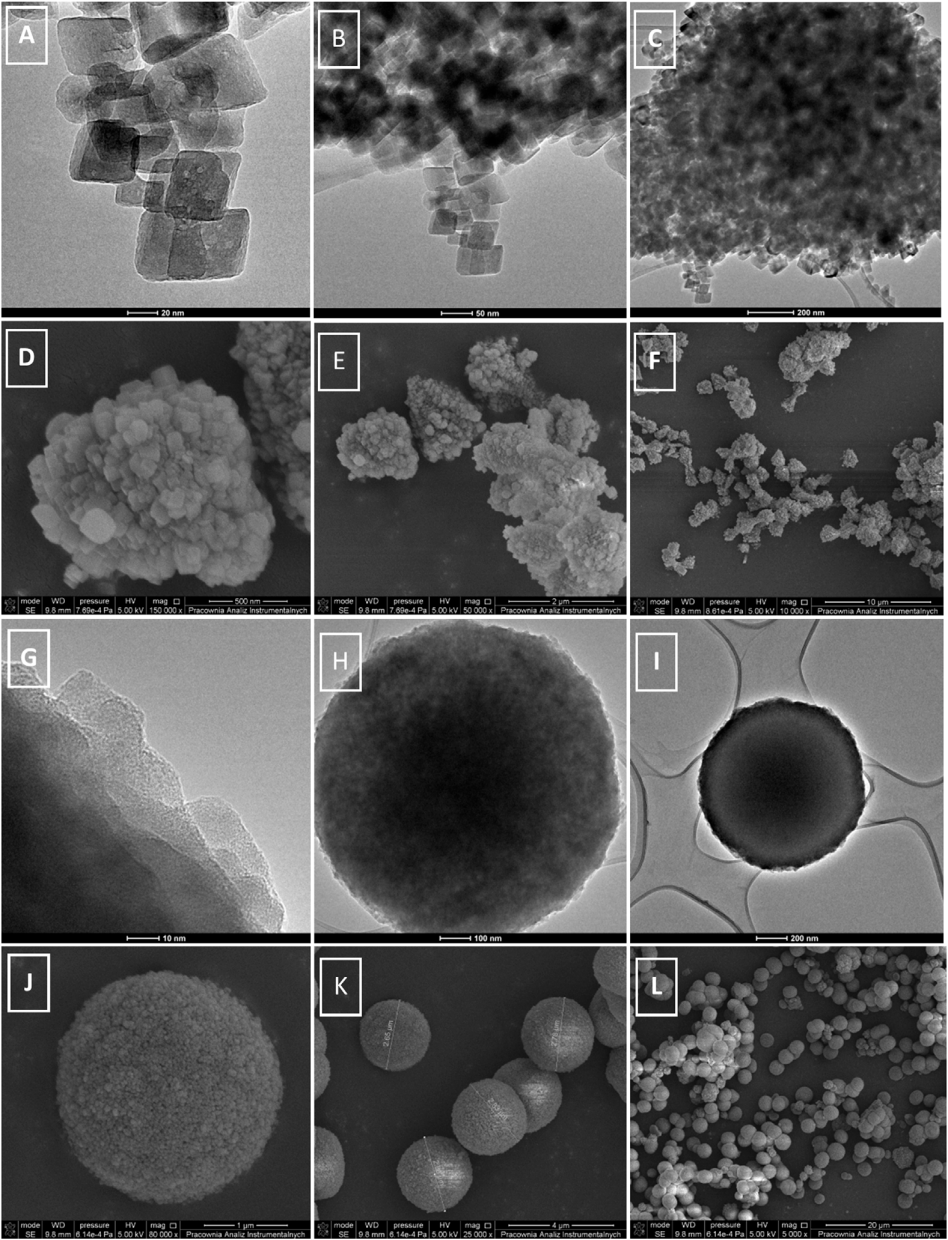
Transmission electron micrograph **(A–C)** and scanning electron micrograph **(D–F)** of CaCO_3_-MS and transmission electron micrograph **(G–I)** and scanning electron micrograph **(J–L)** of sodium caseinate-CaCO_3_-MS.

The synthesis of CaCO_3_-MS by the precipitation method revealed the formation of nanocrystals of CaCO_3_ (Figure 1A) which on nucleation can lead to the formation of the MS (Figure 1). Along the edges of the MS, the rhombohedral nanocrystals of CaCO_3_ calcite crystals are visible, as shown in the TEM micrograph (Figure 1B). The CaCO_3_ nanocrystals and CaCO_3_-MS had an average size of 37.5 nm and 1.28 μm, respectively, as given in the particle size distribution curve (Supplementary Figures S1A and S1B). Although the MS were not perfectly spherical, their overall morphology was consistent.

In contrast, when casein is present, the CaCO_3_ nanocrystals are not visible under TEM; the MS exhibit smooth edges and appear as perfectly spherical structures (Figures 1G–I). TEM analysis revealed that the average diameter of the MS was 2.64 µm and particle size distribution information is given in Supplementary Figure S1C. It seems that the sodium caseinate interacts with the CaCO_3_ crystals, forming larger size perfect MS. There is high demand for perfectly spherical CaCO_3_ particles, especially in the field of oral hygiene, because they offer effective cleaning performance while remaining gentle and non-abrasive (Trushina et al., 2014). The comparable size, number, and density of the MS and casein indicate that individual protein molecules likely initiate nucleation. The protein’s amino acid sequence largely influences the formation of CaCO_3_-MS in the presence of proteins. Amino acids can facilitate nucleation and crystal growth by lowering the activation energy required for nucleation and enhancing crystal development (Briegel et al., 2012). Casein, a milk protein, comprises four peptides, namely, α_s1_, α_s2,_ β, and k-casein, which tend to bind calcium ions, leading to the formation of CaCO_3_-MS; they have a net negative charge on their surface as a result of phosphorylation. Moreover, casein molecules can agglomerate in suitable conditions into spherical micelles (Głąb and Boratyński, 2017). The charged amino acids and some of the amino acids with uncharged polar side chains considerably influenced the CaCO_3_ crystallization. The protein in the presence of the CaCl_2_ due to greater adsorption to the growing crystals would make the crystals more porous and arrange them in spherical form and uniform in size (Vikulina et al., 2018).

Furthermore, Nawarathna et al. (2021) reported that a nucleator protein demonstrates its effectiveness through high nucleation density, a narrow size distribution, and the absence of rhombohedral crystal formation. They have reported using fusion protein (calcite binding peptide and chitin-binding domain) to facilitate the precipitation of CaCO_3_ on the chitin matrix. The protein does act as a template or starting point for the nucleation during the formation of CaCO_3_-MS, and it plays a vital role in the formation of uniform and perfectly spherical CaCO_3_-MS by being absorbed into the growing crystal faces. The casein interacts with Ca^2+^ ions, providing an active site for the initiation of nucleation of CaCO_3_ particles and subsequently adhering to a preferential surface by favoring the growth of spherical CaCO_3_ particles in specific crystallographic planes, enabling the control of the size and stabilizing as spheres. Apart from this, casein can interact with the surface, stabilizing the CaCO_3_-MS and changing properties like hydrophilicity, surface energy, and porosity of the MS. Schematic representation of synthesis of casein-CaCO_3_-MS is given in Supplementary Figure S2.

Presence of citric acid in the reaction mixture also plays an important role in controlling the formation, size, and morphology of CaCO_3_-MS. It also prevents the aggregation of microspheres, which is essential for creating uniform MS (Lucey and Horne, 2018). The equimolar concentration of ammonium carbonate, calcium chloride, and citric acid led to the formation of uniform CaCO_3_-MS by precipitation. In contrast, sodium and potassium carbonate salts could not achieve similar uniformity and spherical MS. However, sodium carbonate is the most extensively studied salt (Liendo et al., 2022). The superiority of ammonium carbonate over sodium and potassium carbonate could be attributed to its gradual decomposition, releasing the ions in a controlled manner, preventing rapid pH changes or better buffering potential, and it is more volatile and diffusible (Frolova et al., 2018).

Changing the reaction conditions, such as stirring time or casein concentration, can also affect CaCO_3_ deposition and formation. In addition, the adsorption rate of the protein casein into the CaCO_3_-MS can be increased by reducing the MS size. The casein concentration of 100, 200, 300, 400, 500, and 1,000 mg in the reaction mixture showed the formation of MS with an average diameter of 5.05, 8.67, 3.00, 5.41, 11.01, and 8.13 µm, respectively (Supplementary Figure S3). One can modify the size of CaCO_3_-MS by adjusting the stirring duration and speed and slowing the salts’ dissolution rate (Parakhonskiy et al., 2014). The mixing time of 5, 10, 15, 30, and 60 min demonstrated the formation of microspheres with average diameters of 10.78, 6.91, 1.37, 1.25 and 1.28 µm, respectively (Supplementary Figure S4). The mixing on a magnetic stirrer mainly affects the mass transfer. Thus, the stirring speed does not significantly affect the crystal size. However, increasing the stirring time reduces the crystal size by enhancing mass transfer and preventing aggregation (Ding et al., 2018). In the present study, we found a stirring time of 30 min at 500 rpm and a casein concentration of 300 mg suitable for forming smaller MS. It may be possible to form even the CaCO_3_ nanosphere if the stirring time increases to a few hours. We observed no change in the size and shape of the samples before and after freeze-drying. Even the MS size and shape did not change after steam sterilization by autoclave at 121°C for 20 min.

**FIGURE 2:**
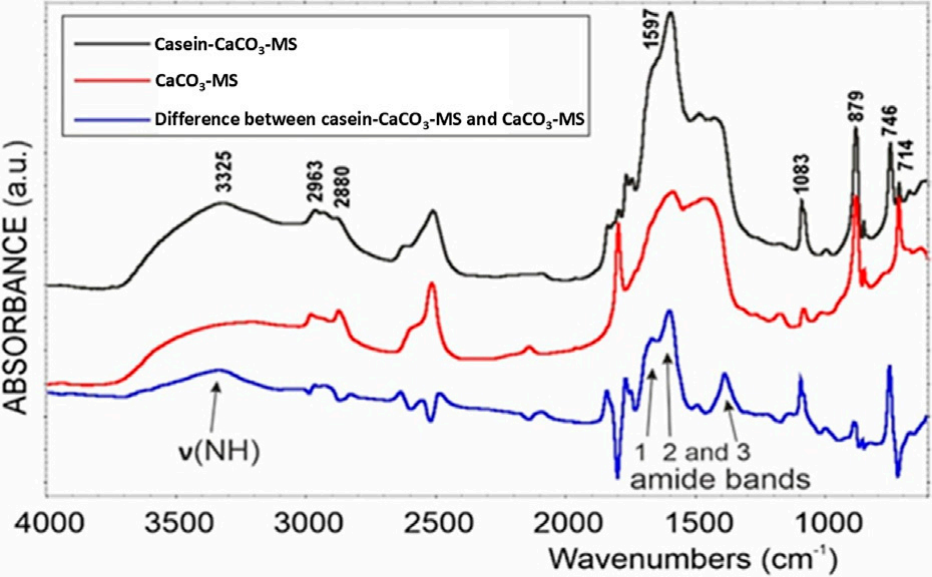
DRIFTS spectra of casein-CaCO_3_-MS, CaCO_3_-MS and difference between casein-CaCO_3_-MS and CaCO_3_-MS.

FTIR analysis of casein-CaCO_3_-MS and CaCO_3_-MS can provide information about their chemical composition, phase structure, and functional groups. DRIFTS spectra of casein-CaCO_3_-MS, CaCO_3_-MS, and their difference showed the presence of amide 1, 2, and 3 linkages, indicating the presence of protein in the structure (Figure 2).

The presence of calcite polymorph in CaCO_3_-MS shows the characteristic peaks due to vibrations in the carbonate ions at 714, 879, 1,083, and 1,402 cm^-1^, whereas the casein CaCO_3_-MS showed the peaks at 746, 879, 1,082, 1,402 and 1,647 cm^-1^ (Supplementary Figure S5). The presence of a band at 746 cm^-1^, a shift in the asymmetric stretching mode, suggests vaterite (Wang et al., 2018), as well as a broadening of the carbonate peak at 1,402 cm^-1^ indicates structural disorder or amorphous calcium carbonate. The XRD and electron microscopy (SEM and TEM) data corroborate the FTIR. Changes in the crystal structure and shape of the particulate nanocrystals prove the strong interaction between the two phases. Moreover, the XRD and electron microscopy (SEM and TEM) results are consistent with the FTIR findings and reveal structural and morphological differences arising from the interaction between casein protein and calcium carbonate.

**FIGURE 3:**
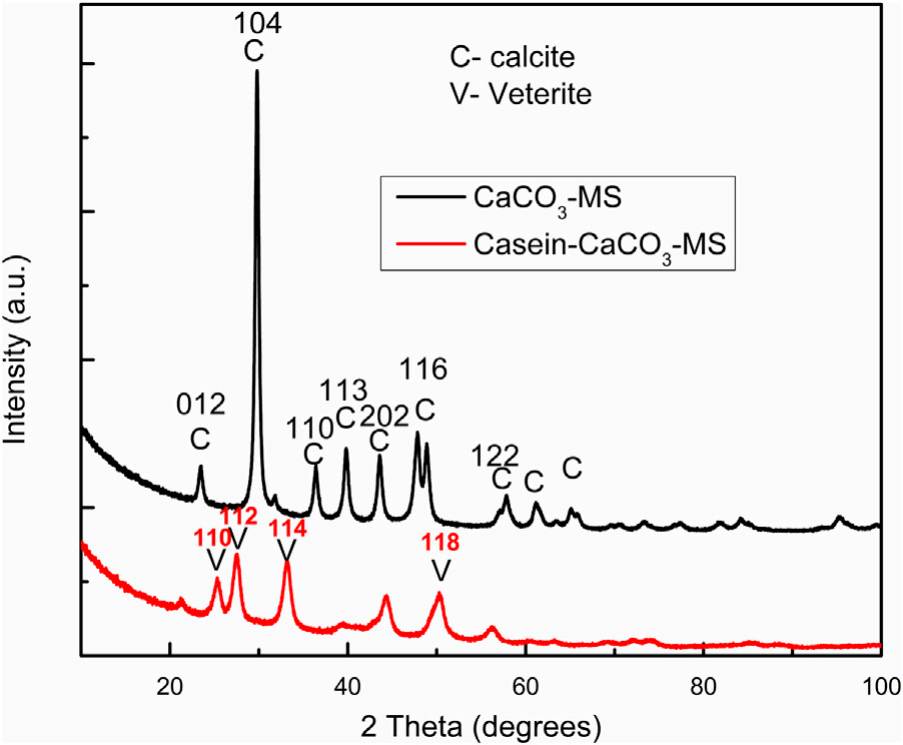
XRD pattern of CaCO_3_-MS and casein CaCO_3_-MS.

The XRD pattern of the CaCO_3_-MS and casein-CaCO_3_-MS is shown in Figure 3. The XRD result in the peaks at 2 theta degree values of 23.02°, 29.35°, 36.0°, 39.4°, 43.2°, 47.3°, 48.6°, 57.5° and 61.3° correspond to the (012), (104), (110), (113), (202), (024), (116), (122) and (119) pure crystallographic planes of rhombohedral calcite crystals (reference code 00-001–0837) without impurities, the result corroborated with the finding of Luo et al. (2020). Whereas the XRD pattern of casein-CaCO_3_-MS shows a mixed pattern of calcite and vaterite, which is in line with the report of Nawarathna et al. (2021). XRD pattern of casein-CaCO_3_-MS (Figure 3) can be corelated with JCPDS card 33–0268 which refers to the standard vaterite pattern. The XRD peaks at 2 theta degree values of 24.9°, 27.0°, 32.8° and 50° correspond to the (110), (112), (114), and (118) crystallographic planes of vaterite crystals (Song et al., 2022). The crystallite size was estimated using the Scherrer equation applied to the XRD diffraction peaks. For the casein-CaCO_3_-MS, the average crystallite size was calculated to be 5.13 ± 0.65 nm, whereas the CaCO_3_-MS exhibited a smaller average size of 3.37 ± 1.26 nm. These results indicate the nanoscale nature of the synthesized materials and suggest that casein incorporation influences the crystallite growth, leading to slightly larger domains compared to the empty matrix. The interaction of calcium ions with the casein protein might be the reason for getting the diffraction pattern for mixed vaterite and calcite form in the casein-CaCO_3_-MS. The MS increased the specific surface area, and the incorporation of casein facilitated the formation of the uniform MS. Lee et al. (2020) have demonstrated the stabilization of cross-linking of carbonyl esterase enzyme in CaCO_3_-MS, which can be used in enzyme-catalyzed reactions involved in bioprocessing, bioconversion, and bioremediation.

The low-temperature N_2_ adsorption measurements and the shape of adsorption isotherm curves indicate the mesoporous character of tested solids with specific surface areas of 65 and 47 m^2^ g^-1^ for casein-CaCO_3_-MS and CaCO_3_-MS, respectively (Supplementary Figure S6). Such low values of specific surface areas and the shape of adsorption isotherms (type II according to the IUPAC classification) indicate that the tested material is rather non-porous. An almost linear increase in adsorption in the relative pressure range of up to 0.9 p/ps, a sudden condensation increases above this value, and small hysteresis loops support this statement. Also, the analysis of the pore distribution (mainly mesopores) proves that the measured porosity comes from the superficial, rough layer, and the larger pores are the result of secondary, intermolecular porosity. Analyzing the pore size distribution also reveals the lack of micropores. We found the smallest pores on the tested surfaces to be larger than 2.6 nm, while the largest exceeded 10 nm. For the casein CaCO_3_-MS, we observed slightly larger pore volumes compared to those of CaCO_3_-MS. For the MS to serve as a carrier for bioactive compounds, an optimal combination of high specific surface area and adequate pore volume ensures that a sufficient amount of bioactive compound can be loaded and released in a controlled manner. The specific surface area enhances the initial loading and surface interactions, while the pore volume governs the rate and profile of the bioactive compound release (Leng et al., 2021).

**FIGURE 4:**
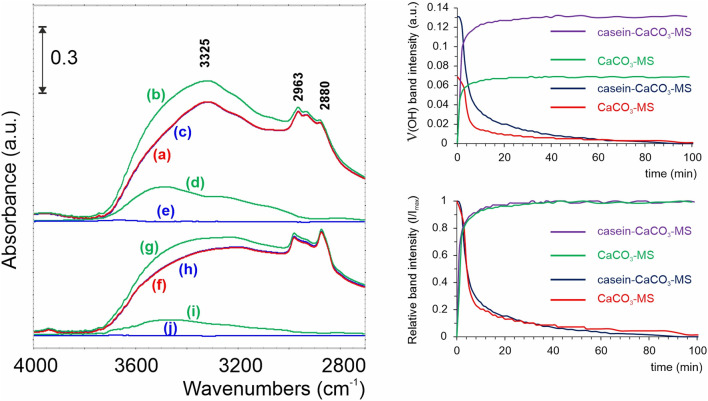
Left panel: spectroscopic investigation of H_2_O adsorption on casein-CaCO_3_-MS and CaCO_3_-MS at 25 °C. (a) casein-CaCO_3_-MS before H_2_O adsorption, (b) casein-CaCO_3_-MS after H_2_O adsorption, (c) casein-CaCO_3_-MS after H_2_O desorption, (d) the differential spectra after H_2_O adsorption and (e) desorption; (f) CaCO_3_-MS before H_2_O adsorption, (g) CaCO_3_-MS after H_2_O adsorption, (h) CaCO_3_-MS after H_2_O desorption; differential spectra after H_2_O adsorption – (i) and desorption at 25 °C – (j); Right panel: time-dependent changes in the ν(OH) band intensities of casein-CaCO_3_-MS and CaCO_3_-MS.

The characteristics of infrared absorption bands related to water molecules adsorbed on the surface, such as O-H stretching and bending vibrations, are typically observed at 3,200 to 3,600 cm^-1^; this broadband corresponds to water molecules’ hydrogen-bonded and free hydroxyl groups. From the results presented in Figure 4, one can see that the adsorption of H_2_O is twice as high for casein-CaCO_3_-MS samples as it is for CaCO_3_-MS ones. The analysis of relative band intensity changes in time demonstrates that both samples have equal adsorption and desorption kinetics. This significant observation shows that casein acts positively by maintaining the H_2_O adsorption/desorption kinetics while simultaneously increasing the adsorption capacity.

Another key finding is that water does not damage the protein structure (Supplementary Figure S7). This means that protein digestion (if needed) will occur in a controlled manner only through proteases or other biological factors, rather than by simple uncontrolled dissolution. This indicates the possibility of the controlled release of drugs, nanoparticles, or nutrient supplements encapsulated in casein-CaCO_3_-MS by protease degradation.

The protein plays an important role in the biomineralization of CaCO_3_ structures. Casein, a milk protein, is generally considered safe (GRAS), biocompatible, and biodegradable. Factors like the type of carbonate salt used, concentration of the protein, and stirring or aging time affect the size and surface properties of the MS formed. In the present study, it is quite evident that the interaction of casein with the CaCO_3_ could lead to the formation of uniform-size MS of calcium carbonate with higher specific surface area, pore volume, and hydrophilic nature, making them a better carrier molecule as compared to CaCO_3_-MS.

## Data Availability

The original contributions presented in the study are included in the article/Supplementary Material, and in the repository under the link: https://repod.icm.edu.pl/dataset.xhtml?persistentId=doi:%2010.18150/OXJ85G further inquiries can be directed to the corresponding author.

## Abbreviations

CaCO_3_-MS, calcium carbonate microspheres; SEM, scanning electron microscopy; TEM, transmission electron microscopy; XRD, X-ray diffraction; DRIFT, diffuse reflectance infrared Fourier transform spectroscopy
