# Supplementary material for: Casein-assisted biomineralization of calcium carbonate microspheres for enhanced surface and adsorption properties
Source: Front Bioeng Biotechnol. 2025 Oct 7;13:1654712. doi: 10.3389/fbioe.2025.1654712 (PMC12537877; doi:10.3389/fbioe.2025.1654712)
Supplement: Supplementary file 1 [file DataSheet1.pdf]

Supplementary Material

Supplementary Figures

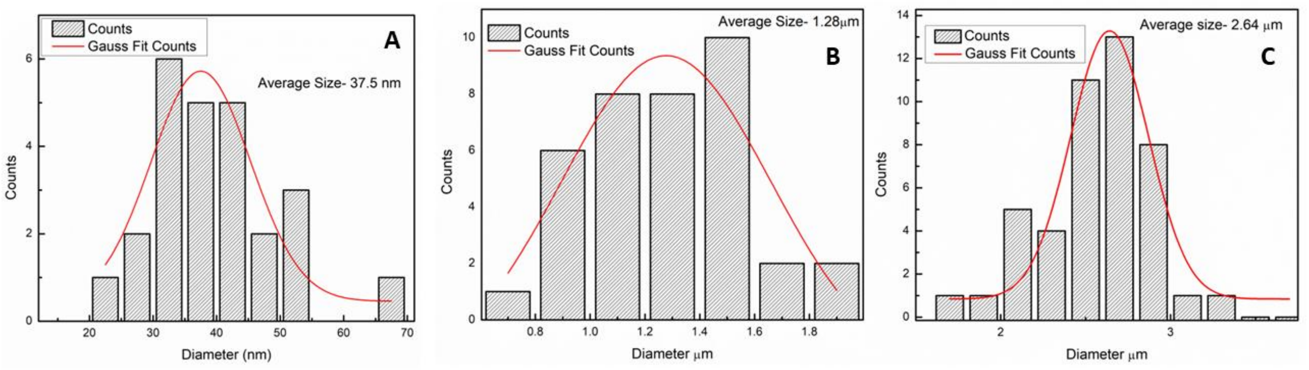

Figure S1 A- Particle size distribution curve of $CaCO_3$ nanocrystals based on Figure 1 (A, B, C), B- Particle size distribution curve of $CaCO_3$-MS based on Figure 1 (D, E, F) and C- Particle size distribution curve of casein-$CaCO_3$-MS based on the Figure 1 (J, K, L).

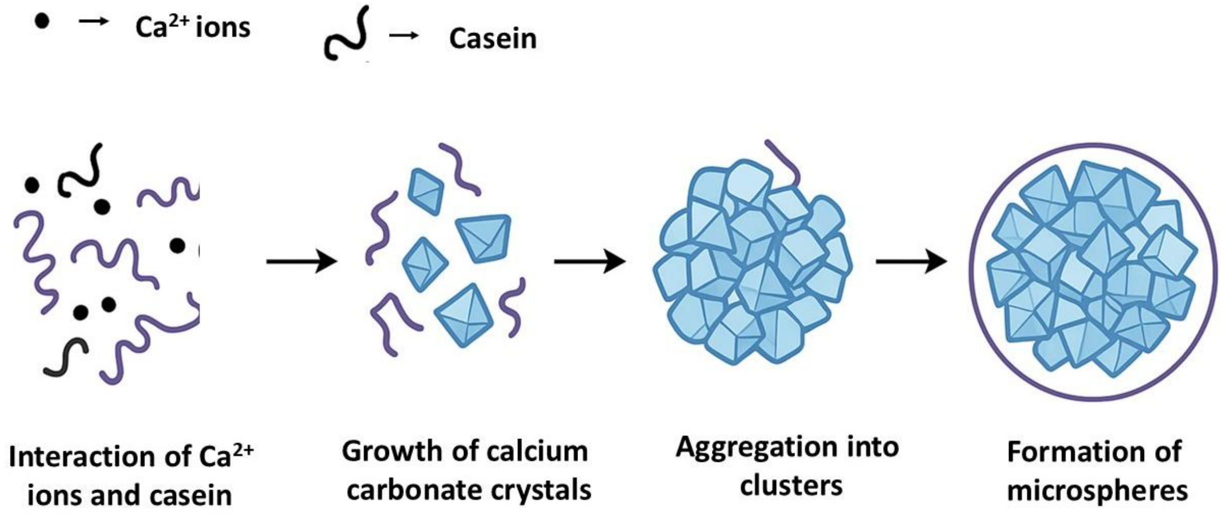

Figure S2 Schematic representation of synthesis of casein-$CaCO_3$-MS

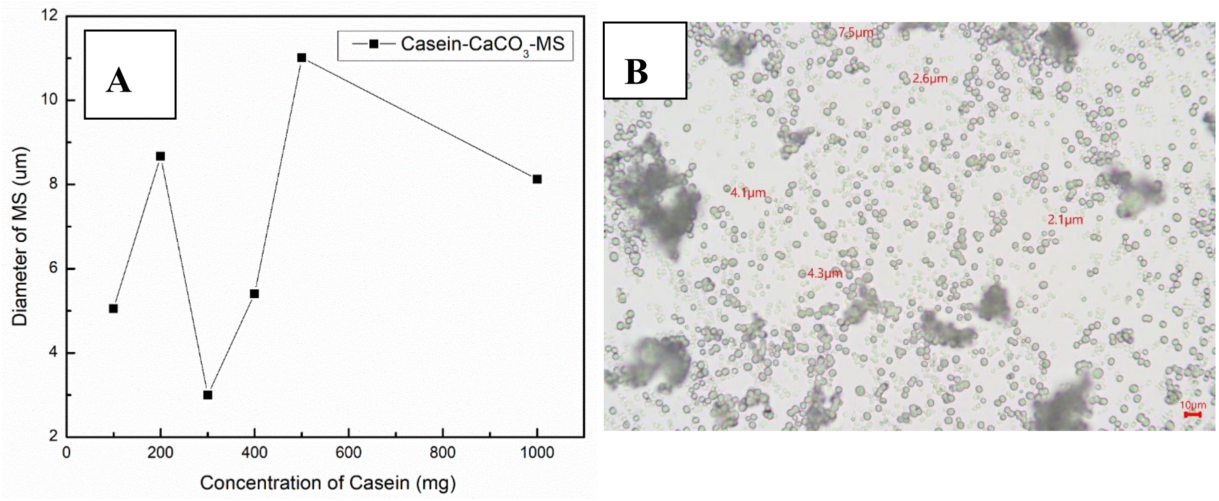

Figure S3 A- Effect of casein concentration on the diameter of MS and B- Optical microscopy image of the microsphere with casein concentration of 300 mg

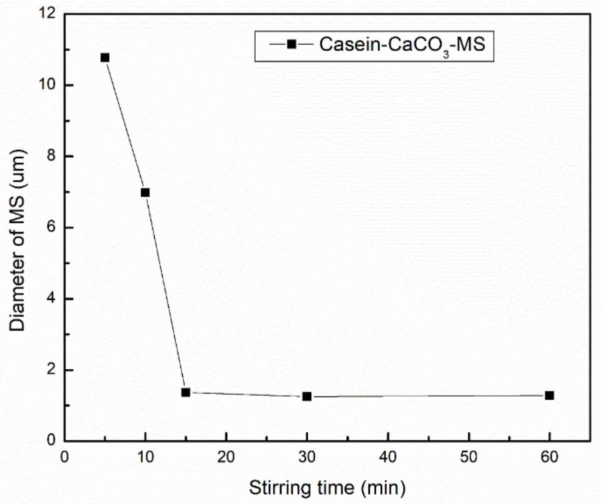

Figure S4 Effect of stirring time on the diameter of MS

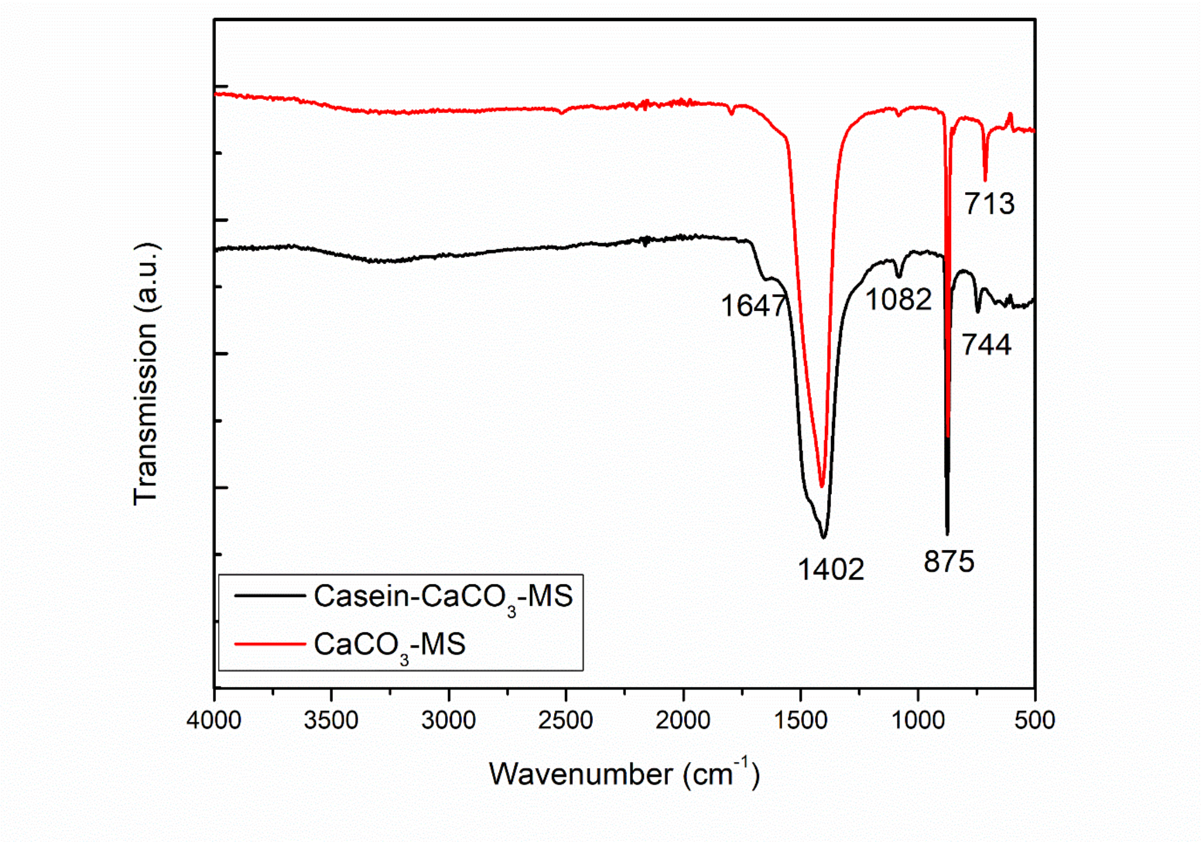

Figure S5 FTIR-ATR spectrum of casein-$CaCO_3$-MS and $CaCO_3$-MS

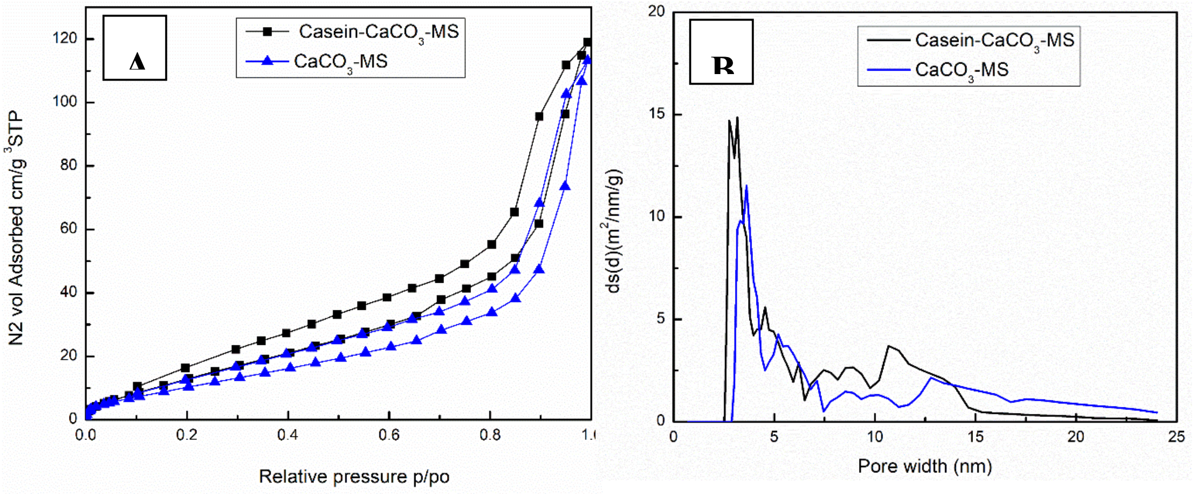

Figure S6 A- N_2 adsorption isotherm and B- Pore width measurement of casein-$CaCO_3$-MS and $CaCO_3$-MS

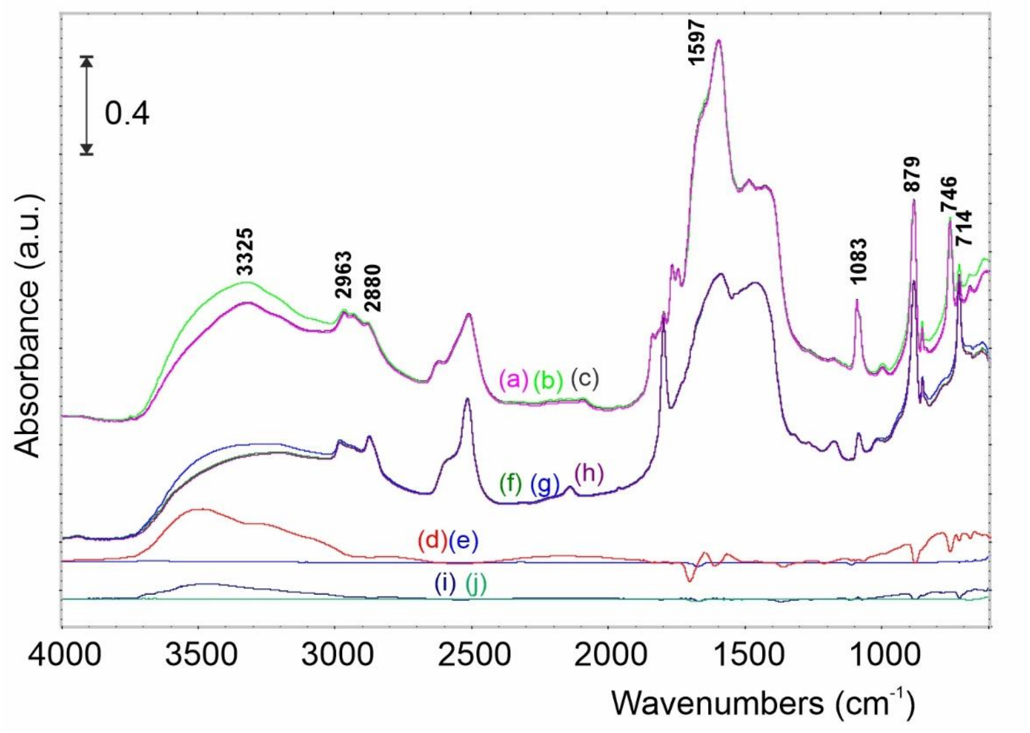

Figure S7 Spectroscopic investigation of H_2O adsorption on casein-$CaCO_3$-MS and $CaCO_3$-MS at 25°C. (a) casein-$CaCO_3$-MS before H_2O adsorption, (b) casein-$CaCO_3$-MS after H_2O adsorption, (c) casein-$CaCO_3$-MS after H_2O desorption; (d) the differential spectra after H_2O adsorption and (e) desorption; (f) $CaCO_3$-MS before H_2O adsorption, (g) $CaCO_3$-MS after H_2O adsorption, (h) $CaCO_3$-MS after H_2O desorption; differential spectra after H_2O adsorption – (i) and desorption at 25°C – (j).
